# Variation of Bone Turnover Markers in Childhood and Adolescence

**DOI:** 10.1155/2023/5537182

**Published:** 2023-07-28

**Authors:** Yiduo Zhang, Jing Zhang, Xiaocui Huang, Xingnan Yu, Ye Li, Fan Yu, Wenjie Zhou

**Affiliations:** ^1^Department of Laboratory Medicine, West China Second University Hospital, Sichuan University, Chengdu, Sichuan, China; ^2^Chengdu Jinjiang District Maternal and Child Healthcare Hospital, Chengdu, China; ^3^Key Laboratory of Birth Defects and Related Diseases of Women and Children (Sichuan University), Ministry of Education, Chengdu, Sichuan, China

## Abstract

**Objectives:**

To determine the bone metabolic marker changes from childhood to adolescence and to provide reference values for monitoring bone development in children in Southwest China.

**Methods:**

We surveyed 703 participants attending physical examinations from April 2019 and August 2021. Twenty-eight participants were excluded for lack of laboratory tests, and 14 people were excluded for diseases that might affect bone metabolism. A total of 661 children were selected for the study. According to the main developmental periods, the children were divided into preschool, preadolescence, and adolescence groups. Serum bone turnover markers including *β*-isomerized C-terminal telopeptide of type I collagen (*β*-CTx), N-terminal midfragment of osteocalcin (N-MID), and procollagen type 1 N-propeptide (P1NP) as well as growth and development indices such as serum calcium (Ca), phosphorus (Pi), alkaline phosphatase (ALP), and vitamin D were measured. The changes in bone metabolism-related markers and the correlations between the indices were analyzed.

**Results:**

During the development in boys, the levels of *β*-CTx and N-MID increased with age from preschool to adolescence, while the levels of P1NP decreased and then increased. In girls, the levels of *β*-CTx and N-MID plateaued in early adolescence and showed little change in subsequent adolescence, while the levels of P1NP exhibited a downward trend. The correlations between bone metabolism markers and vitamin D were not significant.

**Conclusions:**

The levels of bone metabolism markers differed between boys and girls. Reference intervals can be used as essential tools to examine the levels of bone metabolism markers reasonably.

## 1. Introduction

Bone is a primary tissue type in the human body that plays an important role in supporting daily physical and metabolic activities. Bone undergoes dynamic and continuous reconstruction during the lifetime based on osteoblast-dependent bone formation and osteoclast-dependent bone resorption [1]. Dual-energy X-ray absorptiometry and quantitative computed tomography have been used to measure bone mass, providing data on not only bone mass but also bone density and geometry [2, 3]. However, given the potential radiation hazards and examination costs, radiation-based modalities have limited use for long-term monitoring of bone metabolism. Evaluation of biochemical markers can provide different views on the dynamic development of bone tissue and have the potential for more frequent measurements than bone densitometry [3–6]. However, bone turnover markers (BTMs) are mainly focused upon monitoring osteoporosis in menopausal women [6–8], and there remains a lack of corresponding clinical data in children and adolescents during growth and development.

Children and adolescents have significantly elevated levels of bone markers due to their high skeletal growth velocity and rapid bone turnover during childhood growth. However, the stories vary across different stages of bone development and growth. Bone development during childhood and adolescence is an essential determinant of adult skeletal health, and bone metabolism at these developmental stages is more complex than that in adults. Children and adolescents not only require bone reconstruction but also need to maintain linear bone growth.

In many physiological and pathological processes, bone metabolism and bone marker concentration during children's growth will be affected. The establishment of the reference value range of bone markers is helpful in evaluating the bone metabolism of children. Due to different races, regions, and eating habits, there are some differences in children's bone metabolism. Especially in southwest China, where the altitude span is large, and the ethnic groups are complex and diverse, the BTM reference values developed by other laboratories do not apply to children in Southwest China, and it is difficult to assess the bone metabolism of children and adolescents. This study aimed to determine the levels of BTMs in children and adolescents in Southwest China aged 0–16 years, to analyze the changes in BTMs in different growth periods, and to establish reference values for BTMs in different growth periods.

## 2. Materials and Methods

### 2.1. Study Sample

Between April 2019 and August 2021, 703 children and adolescents selected for a health examination at our hospital were evaluated retrospectively as the study subjects. Twenty-eight participants were excluded for lack of laboratory tests, and 14 were excluded for diseases that might affect bone metabolism (6 participants were diagnosed as precocious puberty and 8 were diagnosed as obese). The subjects comprised 366 boys and 295 girls aged 0–16 years. All subjects were free to eat before their examinations. The subjects were divided into preschool (boys and girls aged 0–6 years), preadolescence (boys aged 6–12 years and girls aged 6–10 years), and adolescence (boys aged 12–16 years and girls aged 10–16 years) group according to age. The exclusion criteria were any chronic disease of the skeletal, cardiovascular, or gastrointestinal systems; any chronic disease of the liver, lungs, or kidneys; any hormonal pathology or known process affecting growth and physical development; abnormal bone mineral density; and bone injury in the last three months.

Blood samples were collected in vacuum tubes containing a gel separator and clot activator between 7:00 and 9:00 am after an overnight fast. The samples were allowed to clot for 20 minutes at room temperature and then centrifuged at 1500 × *g* for 10 minutes, and the serum samples were stored at −80°C until analysis.

### 2.2. Patient and Public Involvement

Participants were not involved in the recruitment and conduct of the study. The information of all subjects is obtained from the laboratory information system, registered in the laboratory, and checked on the safety equipment to ensure the absolute safety of patient information. Research involving human subjects complied with all relevant national regulations and institutional policies is in accordance with tenets of the Helsinki Declaration (as revised in 2013)and has been approved by the Ethics Committee of West China Second University Hospital, Sichuan University (reference number: No. 2019033).

### 2.3. Measurement of BTMs and Biochemical Indices

Electrochemiluminescence immunoassays measured the levels of *β*-CTx, N-MID, and P1NP in a Cobas e411 analyzer (Roche Diagnostics GmbH, Mannheim, Germany).

Serum 25(OH) vitamin D levels were determined using a LIAISON 25-OH Vitamin D Total Assay Kit (DiaSorin, Saluggia, Italy), comprising a direct competitive chemiluminescence immunoassay that recognizes 25(OH) vitamin D2 and 25(OH) vitamin D3 and is fully automated using the Liaison platform. Ca, Pi, and ALP were detected by ADVIA 2400 (Siemens, Berlin, Germany). All analytical measurements were performed according to the manufacturer's instructions regarding preventive maintenance, function checks, calibration, and quality control of tests and equipment. All test samples underwent automated interference analysis for hemolysis, hyperbilirubinemia, and lipemia. The manufacturer provided the reagent, quantitative standard, and quality control materials used in the tests. All tests were operated in strict accordance with the instrument and reagent instructions.

### 2.4. Data Analysis

All calculations were performed using SPSS 25.0 software (IBM Corp., Armonk, NY, USA). Data with a normal distribution were shown as mean ± standard deviation. Data with a non-normal distribution were presented as the median and interquartile range (IQR) [25%, 75%]. Data with a non-normal distribution were compared using the Mann–Whitney *U* test or Kruskal–Wallis test. Dunn's multiple comparison test analyzed the differences between the groups. Spearman correlation coefficients were used to estimate the correlations. For reference ranges for the BTMs for the preschool, preadolescence, and adolescence groups, a nonparametric percentile method was applied using 2.5 to 97.5 percentiles with a 90% confidence interval (CLSI C28-A3). Values of *P* < 0.05 were considered significant for all tests. For visualization, figures were drawn with ggplot2 of R language (http://www.r-project.org/).

## 3. Results

### 3.1. Changes of BTMs in Different Age Groups

In Table 1, in boys aged 0–16 years, the levels of P1NP decreased with chronological age up to the preadolescence group and then increased. A significant difference was observed between preschool (*P* < 0.001) and adolescence (*P* = 0.007). Meanwhile, the levels of *β*-CTx and N-MID increased with age in boys. There were significant differences in these levels between preschool and both preadolescence (*P* < 0.001 and *P* = 0.006) and adolescence (*P* < 0.001 and *P* < 0.001). There were also significant differences in these levels between preadolescence and adolescence (*P* < 0.001 and *P* < 0.001).

In girls, a downward trend in the levels of P1NP was observed from preschool through adolescence. The level of P1NP in preschool was significantly higher than that in adolescence (*P* = 0.012). Meanwhile, the levels of *β*-CTx in preschool were significantly lower than those in preadolescence (*P* = 0.043) and adolescence (*P* = 0.01). Similarly, the levels of N-MID in preschool were significantly lower than those in preadolescence (*P* < 0.001) and adolescence (*P* < 0.001).

The levels of 25(OH) vitamin D decreased with age in both boys and girls. In boys and girls, the levels of 25(OH) vitamin D in preschool were higher than those in preadolescence (*P* < 0.001 and *P* < 0.001) and adolescence (*P* < 0.001, *P* < 0.001).

### 3.2. Levels of BTMs in Different Sexes during the Same Growth Period

**Figure 1:**
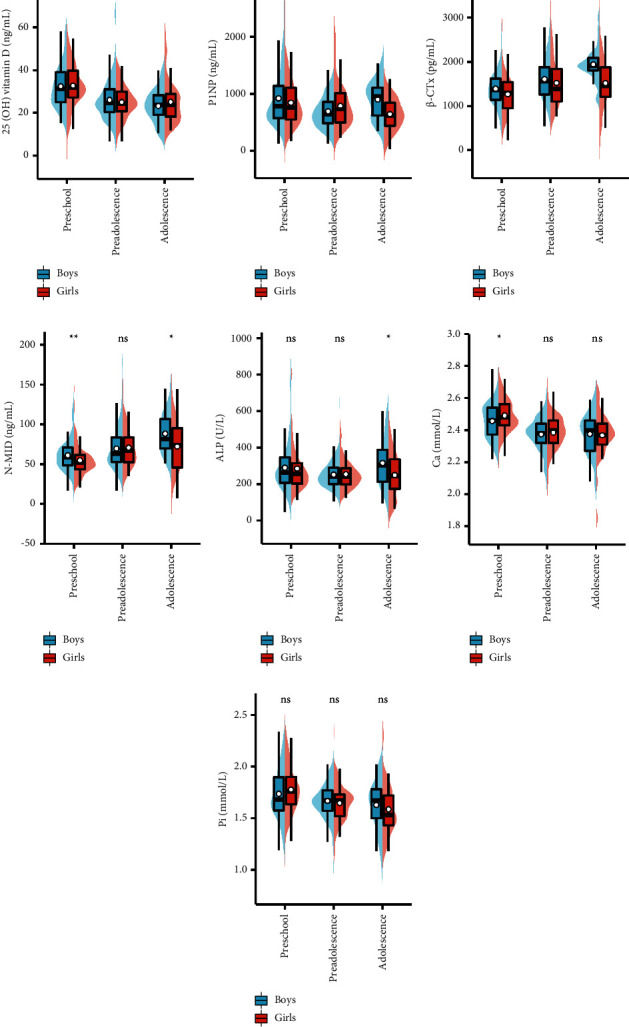
Comparison of BTM levels between boys and girls in different growth periods. ns, *P *≥* *0.05; ^*∗*^*P* < 0.05; ^*∗∗*^*P* < 0.01; ^*∗∗∗*^*P* < 0.001.

There were no significant differences in the levels of 25(OH) vitamin D (Figure 1(a)) between boys and girls in the different age groups. The levels of P1NP in boys were higher than that in girls in adolescence (*P* = 0.024) (Figure 1(b)). The levels of *β*-CTx in boys were higher than those in girls in preschool and adolescence (*P* = 0.014 and *P* < 0.001) (Figure 1(c)), where the levels of N-MID (*P* = 0.002 and *P* = 0.016) (Figure 1(d)). The levels of ALP in boys were higher than those in girls in adolescence (*P* = 0.014) (Figure 1(e)). The levels of Ca in girls were higher than that in boys in preschool (*P* = 0.012) (Figure 1(f)). There were no significant differences in the levels of Pi (Figure 1(g)) between boys and girls in the different age groups.

### 3.3. Correlation between BTMs and Age

**Figure 2:**
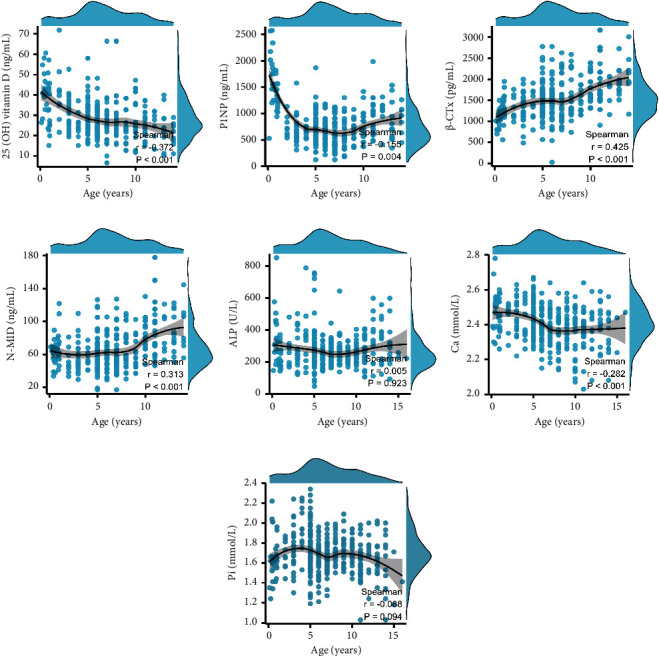
Correlations between BTMs and age in boys. (a) 25 (OH) vitamin D and age. (b) P1NP and age. (c) *β*-CTX and age. (d) N-MID and age. (e) ALP and age. (f) Ca and age. (g) Pi and age.

In boys, P1NP and Ca were weak and negatively correlated with age (Figures 2(b) and 2(f)), 25(OH) vitamin D was moderately negatively correlated with age (Figure 2(a)), while *β*-CTx and N-MID were moderately positively correlated with age (Figures 2(c) and 2(d)). ALP and Pi were negligibly correlated with age (Figures 2(e) and 2(g)).

**Figure 3:**
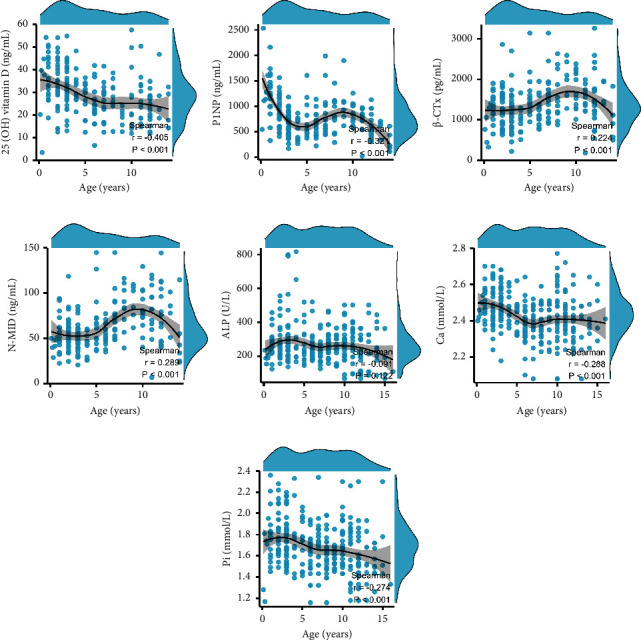
Correlations between BTMs and age in girls. (a) 25 (OH) vitamin D and age. (b) P1NP and age. (c) *β*-CTX and age. (d) N-MID and age. (e) ALP and age. (f) Ca and age. (g) Pi and age.

In girls, Ca and Pi were weak and negatively correlated with age (Figures 3(f) and 3(g)), 25 (OH) vitamin D and P1NP were moderately negatively correlated with age (Figures 3(a) and 3(b)), while *β*-CTx and N-MID were weak and positively correlated with age (Figures 3(c) and 3(d)). ALP was negligibly correlated with age (Figure 3(e)).

### 3.4. Relationship of BTMs and Development Indices

**Figure 4:**
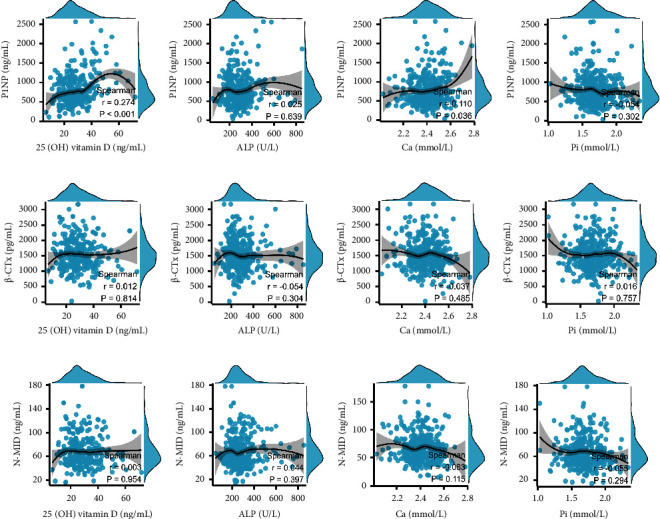
Scatterplots showing the correlations between serum P1NP, N-MID, and *β*-CTX levels and development indices in boys aged 0–16 years. (a) P1NP and 25 (OH) vitamin D (b) P1NP and ALP. (c) P1NP and Pi. (d) P1NP and Ca. (e) N-MID and 25 (OH) vitamin D. (f) N-MID and ALP. (g) N-MID and Pi. (h) N-MID and Ca. (i) *β*-CTX and 25 (OH) D. (j) *β*-CTX and ALP. (k) *β*-CTX and Pi. (l) *β*-CTX and Ca.

In boys, the P1NP levels were weak and positively correlated with 25 (OH) vitamin D (Figure 4(a)) and Ca (Figure 4(c)). There was negligible difference between BTMs and other development indicators (Figures 4(b)–4(l)).

**Figure 5:**
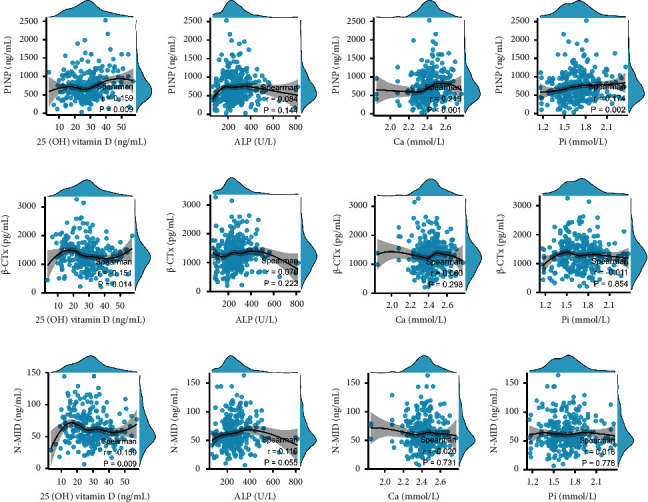
Scatterplots showing the correlations between serum P1NP, N-MID, and *β*-CTX levels and development indices in girls aged 0–16 years. (a) P1NP and 25 (OH) vitamin D. (b) P1NP and ALP. (c) P1NP and Pi. (d) P1NP and Ca. (e) N-MID and 25 (OH) vitamin D. (f) N-MID and ALP. (g) N-MID and Pi. (h) N-MID and Ca. (i) *β*-CTX and 25 (OH) D. (j) *β*-CTX and ALP. (k) *β*-CTX and Pi. (l) *β*-CTX and Ca.

In girls, the P1NP levels were weak and positively correlated with 25 (OH) vitamin D (Figure 5(a)) and Ca (Figure 5(c)). The *β*-CTX and N-MID levels were weak and negatively correlated with 25 (OH) vitamin D (Figures 5(e) and 5(i)). There was negligible difference between BTMs and other development indicators (Figures 5(b), 5(d), 5(f), 5(g), 5(h), and 5(j)–5(l)).

### 3.5. Biomedical Reference Ranges for BTMs

Depending on the analyte in question, 661 children and adolescents were tested for BTMs examined, and the results were used to calculate age- and sex-specific reference intervals. The information regarding the reference intervals is shown in Table 2.

## 4. Discussion

Bone mineralization during childhood and adolescence establishes the foundation for bone health throughout life because 90% of bone mass accrual occurs during the first 20 years [9]. Healthy bone development requires intricately balanced endocrine and mineral metabolism [10]. Because childhood and adolescence are the most critical periods for bone development, monitoring of bone metabolism, and skeletal development during childhood and adolescence is becoming increasingly important.

P1NP and C-terminal propeptide of type I procollagen (PICP) are bone formation markers that are synthesized from procollagen precursors [11]. Specific proteases remove them during the conversion of procollagen to collagen and its embedding in the bone matrix [12]. P1NP has a longer circulating half-life*in vivo* than PICP and is used as a specific marker for bone formation and osteoblast activity. The serum level of P1NP is directly related to the amount of newly formed collagen in bone [5]. The International Osteoporosis Foundation and the International Federation of Clinical Chemistry and Laboratory Medicine recommend that serum P1NP should be employed as a reference marker for bone formation [13]. From preschool to preadolescence, the levels of P1NP were observed to decrease and then only moderately increase toward puberty. These results were consistent with those in a previous report [14]. The trend for P1NP in girls differed from that in boys. In girls, high levels of P1NP remained during preschool and preadolescence, while the levels of P1NP decreased and moderately fluctuated until adolescence. Regarding differences related to sex, our study showed that the changes in P1NP in boys were delayed to a certain extent compared with those in girls, which can be connected to differences in the development of endocrine changes and growth velocities between the sexes [15, 16]. Girls tend to reach the peak of growth and development before first menstruation, while boys show testicular enlargement and subsequent penis growth as the first physical signs of puberty, followed by peak growth [17]. Compared with girls, the rapid growth of boys during puberty occurs later. Therefore, the levels of P1NP in boys are higher than that in girls during puberty.

*β*-CTx is released into the blood by the degradation of type I collagen during bone resorption by osteoclasts. It is a sensitive marker that reflects osteoclast activity and is internationally recognized as a marker for bone resorption [18]. There were different change trends for *β*-CTx in girls and boys. In boys from preschool through adolescence, the levels of *β*-CTx increased with advancing age, and there was a positive correlation between age and *β*-CTx. In girls, the level of *β*-CTx increased from preschool to preadolescence, did not change markedly from preadolescence to adolescence, and instead seemed to reach a plateau. Sex differences in bone development were apparent and related to puberty development and the influence of the bone size. Because of the earlier skeletal maturation, the degree of bone resorption reached a plateau in girls compared with boys.

The changes in N-MID levels were almost consistent with the changes in *β*-CTx levels in both girls and boys. N-MID is a stable osteocalcin carboxyl fragment secreted by mature osteoblasts that can precisely reflect the status of bone formation, including the activity of osteoblasts and the rate of bone mineralization [19]. Serum osteocalcin is regarded as a marker for the activity status of osteoblasts, mainly newly formed osteoblasts. Bone growth and development is a dynamic process maintained by a balance between bone formation and resorption. Among boys and girls, the change trends in N-MID were different, which may arise from the different degrees of development among the sexes. In adolescence, boys have more prominent and broader bones than girls, and their bone metabolism is more active.

Vitamin D plays a crucial role in calcium metabolism and is essential for bone health in adolescents because of its contribution to bone development [10]. In the present study, vitamin D levels in boys and girls showed a downward trend with increasing age. Thiering [20] observed lower *β*-CTx levels in children with higher circulating vitamin D levels and a decreased *β*-CTx-to-osteocalcin ratio. Although no significant correlations were found between vitamin D levels and BTMs, we still observed opposite trends between vitamin D and *β*-CTx. A possible reason for the lack of correlation between vitamin D levels and BTMs may be the insufficient number of subjects with vitamin D deficiency. Therefore, the correlations between vitamin D levels and BTMs warrant further investigation. However, the present findings at least suggest that there will be a loss or deficiency of vitamin D during the process of growth and development in children, implying the need for timely supplementation at this stage to prevent the onset of growth and development disorders.

The correlations between BTMs and biochemical indices such as vitamin D, ALP, and calcium have remained unclear. Increased vitamin D levels in adults result in lower bone turnover, while bone metabolism remains stable. During development in children, bone metabolism is more complicated than adults because bone acquisition and remodeling occur, especially during active growth in puberty. When the vitamin D level was 75 nmol/L in puberty, bone marker levels were significantly elevated [21]. In the present study, vitamin D levels were consistent with the levels of BTMs in girls. Vitamin D levels were also correlated with P1NP levels in boys. However, we found that the correlations between BTMs and calcium and phosphorus levels were insignificant. This may arise through compensation by ions in the body, required to maintain calcium and phosphorus levels at relatively normal levels and ensure normal physiological activities.

There is some limitation in our research. Firstly, there is a lack of multicenter data. From low altitude to high altitude areas, the differences between different regions and ethnicities have not been studied. This will be implemented in our following research. Secondly, each laboratory's research population is different, so the reference value application needs further verification.

## 5. Conclusions

In conclusion, biochemical BTMs were measured in boys and girls aged 0–16 years, to determine the nature of the changes in serum markers of bone metabolism and how they were related to the processes of natural growth and development in children. We have defined reference values for Southwest China and provided effective monitoring and evaluation tools for growth and development assessment of children from preschool to adolescence.

## Figures and Tables

**Table 1:** Bone metabolism indices and basic indices during growth in children.

		Age (years)	25(OH) vitamin D (ng/mL)	P1NP (ng/mL)	*β*-CTx(pg/mL)	N-MID (ng/mL)	ALP(U/L)	Ca (mmol/L)	P_i_(mmol/L)
Boys (*n* = 366)	Preschool (*n* = 142)	3 (1, 5)	31. 6 (27.1, 38.8)	798 (577, 1241)	1350 (1140, 1620)	59.8 (48.7, 71.7)	263.5 (207, 348)	2.46 (2.37, 2.54)	1.68 (1.58, 1.90)
	Preadolescence (*n* = 183)	8 (7, 10)	24.4 (18.5, 30.1)^*∗*^	644 (483, 886)^*∗*^	1550 (1250, 1880) ^*∗*^	63.4 (52.8, 83.9)^*∗*^	242 (201, 291)	2.38 (2.32, 2.44)^*∗*^	1.67 (1.57, 1.77)^*∗*^
	Adolescence (*n* = 41)	13 (12, 14)	24.3 (18.6, 28.9)^*∗*^	755 (502, 1058)	1850 (1283, 1990)∗	78.5 (56.9, 106.3)^*∗*^^†^	312 (208, 467)^†^	2.41 (2.27, 2.47)^*∗*^	1.69 (1.62, 1.77)^*∗*^

Girls (*n* = 295)	Preschool (*n* = 124)	3 (2, 3)	31.6 (27.1, 38.8)	767 (552, 1150)	1290 (923, 1540)	52.1 (43.7, 61.7)	257 (205, 324)	2.48 (2.43, 2.56)	1.76 (1.64, 1.9)
	Preadolescence (*n* = 91)	7 (6, 8)	24.4 (18.5, 30.1)^*∗*^	728 (490, 1024)^*∗*^	1385 (1089, 1878)	67.2 (52.0, 84.0)^*∗*^	240 (199, 287)	2.4 (2.32, 2.46)^*∗*^	1.67 (1.52, 1.73)^*∗*^
	Adolescence (*n* = 80)	12 (11, 12)	24.3 (18.6, 28.9)^*∗*^	580 (401, 788)^*∗*^	1303 (972, 1720)	62.3 (43.2, 85.9)^*∗*^	244 (174, 336)	2.37 (2.31, 2.44)^*∗*^	1.53 (1.43, 1.72)^*∗*^

Note: ^*∗*^compared with the preschool group, *P* < 0.05; ^†^compared with the preadolescence group, *P* < 0.05.

**Table 2:** Biomedical reference ranges for BTMs in children.

		Age, years	2.5th percentile	97.5th percentile	90% CI for low limit	90% CI for high limit
P1NP (ng/mL)	Boys	0–6	266.5	2246.3	87.4–374.6	1864.1–2570.2
		6–12	221.4	1298.8	175.2–273.3	1175.0–1419.0
		>12	117.6	1532.1	106.8–350.4	1308.1–1537.0
	Girls	0–6	268.8	1935.0	182.1–354.9	1605.0–2473.6
		6–10	230.9	1809.0	30.5–286.5	1141.0–1987.0
		>10	97.2	1130.1	26.3–136.1	1034.8–1260.0

*β*-CTx (pg/mL)	Boys	0–6	560.3	2346.5	395.7–754.3	1994.2–2630.0
		6–12	796.0	2760.0	542.0–859.0	1940.0–3096.0
		>12	663.6	2996.0	663.3–898.2	2210.0–3010.0
	Girls	0–6	444.2	2678.3	245.1–613.2	1960.0–3096.0
		6–10	485.0	2618.0	180.5–637.8	2379.0–3140.0
		>10	211.2	2585.7	182.0–426.9	2270.0–3260.0

N-MID (ng/mL)	Boys	0–6	24.9	117.5	16.1–33.4	105.0–121.8
		6–12	31.6	138.7	28.9–36.9	118.6–177.9
		>12	19.0	143.6	18.6–36.7	120.7–144.7
	Girls	0–6	24.7	125.6	21.1–27.0	93.4–161.9
		6–10	27.8	114.6	9.9–37.7	106.9–144.6
		>10	17.4	129.1	6.8–22.3	114.5–144.2

ALP (U/L)	Boys	0–6	104	753	62–129	574–803
		6–12	140	402	106–147	368–443
		>12	93	599	92–125	554–599
	Girls	0–6	143	818	121–155	565–1952
		6–10	144	460	144–161	383–467
		>10	65	502	65–83	457–502

Ca (mmol/L)	Boys	0–6	2.26	2.66	2.24–2.28	0.261–2.72
		6–12	2.13	2.58	2.07–2.15	2.53–2.64
		>12	2.08	2.59	2.08–2.22	2.54–2.59
	Girls	0–6	2.30	2.70	2.18–2.33	2.66–2.72
		6–10	2.10	2.60	2.08–2.23	2.51–2.64
		>10	1.85	2.65	1.85–2.08	2.61–2.65

P_i_ (mmol/L)	Boys	0–6	1.26	2.44	1.21–1.34	2.18–2.30
		6–12	1.32	1.98	1.25–1.41	2.53–2.64
		>12	1.03	2.02	1.03–1.24	1.99–2.02
	Girls	0–6	1.39	2.28	1.17–1.44	2.20–2.36
		6–10	1.16	1.98	1.16–1.36	1.92–1.98
		>10	1.18	2.30	1.18–1.28	2.01–2.30

25(OH) vitamin D (ng/mL)	Boys	0–6	16.2	58.0	15.1–17.4	53.6–64.6
		6–12	11.7	47.6	8.6–12.7	42.2–66.4
		>12	8.8	39.8	8.8–11.0	33.9–39.9
	Girls	0–6	9.5	54.2	3.5–14.0	48.3–54.9
		6–10	9.5	43.6	6.6–12.2	38.7–53.4
		>10	11.6	52.0	10.8–13.0	47.2–57.6

## Data Availability

The data used to support the findings of this study are included within the article.

## Abbreviations

*β*-CTx: *β*-Isomerized C-terminal telopeptide of type I collagen
N-MID: N-terminal midfragment of osteocalcin
P1NP: Procollagen type 1 N-propeptide
ALP: Alkaline phosphatase
BTMs: Bone turnover markers
IQR: Interquartile range
PICP: C-terminal propeptide of type I procollagen.
